# A Machine Learning Methodology for Identification and Triage of Heart Failure Exacerbations

**DOI:** 10.1007/s12265-021-10151-7

**Published:** 2021-08-28

**Authors:** James Morrill, Klajdi Qirko, Jacob Kelly, Andrew Ambrosy, Botros Toro, Ted Smith, Nicholas Wysham, Marat Fudim, Sumanth Swaminathan

**Affiliations:** 1 Department of Mathematics, University of Oxford, Oxford, UK; 2 Booking.com, Amsterdam, Netherlands; 3 Alaska Heart, Anchorage, AK USA; 4 Department of Cardiology, Kaiser Permanente San Francisco Medical Center, San Francisco, CA USA; 5 Division of Research, Kaiser Permanente Northern California, Oakland, CA USA; 6 Vironix Health, Austin, TX USA; 7 Department of Medicine, University of Louisville, Louisville, KY USA; 8 The Vancouver Clinic, Vancouver, WA USA; 9 Washington State University School of Medicine, Spokane, WA USA; 10 Division of Cardiology, Duke University Medical Center, Durham, NC USA; 11 Duke Clinical Research Institute, Durham, NC USA; 12 Industrially Focused Mathematical Modelling Center for Doctoral Training, University of Oxford, England, UK

**Keywords:** Congestive heart failure, Early detection and treatment, Telehealth monitoring, Machine learning, Exacerbation, triage, Triage

## Abstract

**Abstract:**

Inadequate at-home management and self-awareness of heart failure (HF) exacerbations are known to be leading causes of the greater than 1 million estimated HF-related hospitalizations in the USA alone. Most current at-home HF management protocols include paper guidelines or exploratory health applications that lack rigor and validation at the level of the individual patient. We report on a novel triage methodology that uses machine learning predictions for real-time detection and assessment of exacerbations. Medical specialist opinions on statistically and clinically comprehensive, simulated patient cases were used to train and validate prediction algorithms. Model performance was assessed by comparison to physician panel consensus in a representative, out-of-sample validation set of 100 vignettes. Algorithm prediction accuracy and safety indicators surpassed all individual specialists in identifying consensus opinion on existence/severity of exacerbations and appropriate treatment response. The algorithms also scored the highest sensitivity, specificity, and PPV when assessing the need for emergency care.

**Lay summary:**

Here we develop a machine-learning approach for providing real-time decision support to adults diagnosed with congestive heart failure. The algorithm achieves higher exacerbation and triage classification performance than any individual physician when compared to physician consensus opinion.

**Graphical abstract:**

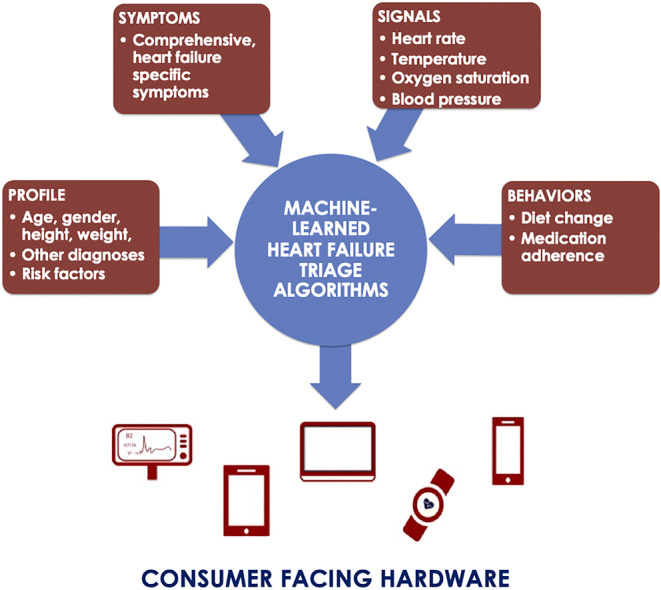

**Supplementary Information:**

The online version contains supplementary material available at 10.1007/s12265-021-10151-7.

## Introduction

Heart failure (HF) is a chronic, progressive condition in which the heart muscle is unable to pump sufficient blood to meet the body’s circulatory and oxygenation needs. At present, the global prevalence of HF is estimated to include 38 million people [1] with an associated direct and indirect cost burden of $108 billion [2]. Hospitalizations from HF exacerbations account for 6.5 million hospital days, the leading cause of hospitalization in the USA and Europe [3]. Acute exacerbations of HF are due to a maladaptive accumulation of intravascular volume eventually leading to dyspnea and respiratory failure but can be identified by changes in symptoms and physiologic parameters. If recognized in a timely manner, exacerbations can be safely managed at home through changes to medication and diet. Despite the recognized impact of these exacerbations, there is no universally accepted clinical approach for self-identification of HF exacerbations by patients at home. Patients are often unaware of both the onset and severity of symptom escalations due to the inherent variability in daily disease management. The result is delayed treatment (which leads to more severe exacerbation episodes), long-term debilitation in quality-of-life, and severe economic burden. Early recognition and treatment of HF exacerbations is an unmet need that leads to morbidity, mortality, and unnecessary healthcare utilization.

Currently, the gold standard for at-home patient self-management of HF is defined as “daily activities that maintain clinical stability” [4]. This requires that patients monitor their medication, diet, exercise, and symptoms by recognizing changes and responding on their own [5]. There are also sparsely used disease “action plans” where the patient refers to a document when feeling concerned about his or her symptoms. These documents generally include color-coded deterministic, condition-based guidelines that direct a patient to either continue with their treatment as normal, alter treatment in a prescribed fashion, call a physician, or go to the emergency room depending on the severity of presented symptoms [6, 7].

While the medical guidance in these checklists has demonstrated utility in patient education, the simple decision tree-like structure of these approaches does not effectively capture the interactions between different patient health state parameters during a HF exacerbation. Furthermore, most current action plans focus heavily on response to rapid weight gain, which further demonstrates the need for improved methodologies for assessing the current state of patient health. Machine-learning methods, by contrast, are more effective in capturing complex inter-dependencies between health variables when making predictions on various health deterioration events [8–10].

Here, we report on the development and validation of a triage methodology that utilizes machine learning methods for identifying HF exacerbations, assessing their severity, and providing an associated care recommendation. Recommendations are made based on baseline patient profile data and one-time responses to symptom and vital sign factors. Validation data consists of a 100-case validation set with ground truth labels corresponding to the consensus opinion of 8 board-certified medical professionals. Algorithm performance on this consensus set is compared against the performance of the individual doctor triage recommendations against the consensus.

## Methods

### Data Generation

The process of generating cases, training, and validating follows closely with the methods explained in detail in [9]. Here, we include a summary of the process.

Physician input was used to facilitate three major aspects of the algorithm development process:

1. Algorithm feature (clinical variable) selection
2. Algorithm training data
3. Algorithm validation data

All participating physicians were board-certified physicians or physician assistant triage specialists from both private and academic institutions. The detailed list of each specialist and his/her credentials is indicated in Table 4. The collective pool included a diverse set of age, gender, race/ethnicity, and practice type/location considerations.

The data consisted of a training set of 1900 patient scenarios with baseline, vitals, and symptoms data. Of critical importance when developing these scenarios was to include the relevant sign, symptom, and profile information that could reasonably be captured by patients at home through either patient input or commonly used devices/databases (rather than blood biomarker or clinical lab data). A random selection of 100 cases was chosen for the validation set and the remaining 1800 as the training (one of the validation cases was removed in the analysis due to physician agreement on its clinical in-feasibility). The training set was shuffled and sent to a group of six specialists to provide opinions on the severity of the patients’ profile, symptoms, and vitals as well as a decision on exacerbation, triage, and recommended treatment. An additional two specialists gave their opinions on only the validation set so as to introduce a subset of validation case scorers who made no contribution to algorithm training.

The scoring process for patient cases was done as a spreadsheet exercise with scorers providing expert opinion in a manner similar to the assessment of a patient vignette. Case scorers were presented with baseline and current health data for patients having a minimum diagnosis of HF. Vital sign and symptom data also included uncertainty to capture scenarios in which a patient is either unable to enter data (does not have the necessary equipment/device) or truly uncertain about the answer (for example, when a patient cannot say whether their chest pain is really different from usual). The specialists examined the case scenarios and provided the following input labels on each case.

1. Profile, symptom, and vitals features each ranked from 1 to 5 (least severe to most severe).
2. Exacerbation assessment, **Yes** or **No**.
3. Triage value of 1–4 where
  i. **Okay**—No additional treatment required.
  ii. **Plan**—Continue your medication plan as normal and check back in 1–2 days.
  iii. **Doctor**—Call your physician.
  iv. **ER**—Go to the emergency room.
4. Recommended treatment score 1–5 where
  i. No additional treatment required
  ii. Double (or increase) dose of your loop diuretic
  iii. Double dose of your loop diuretic and take an additional afternoon dose. Schedule an appointment with your provider to follow-up
  iv. Schedule an appointment with your doctor as you may need IV diuretics. This should be evaluated by your heart management center or provider.
  v. I am not comfortable making a treatment recommendation without seeing the patient in person.

**Figure 1:**
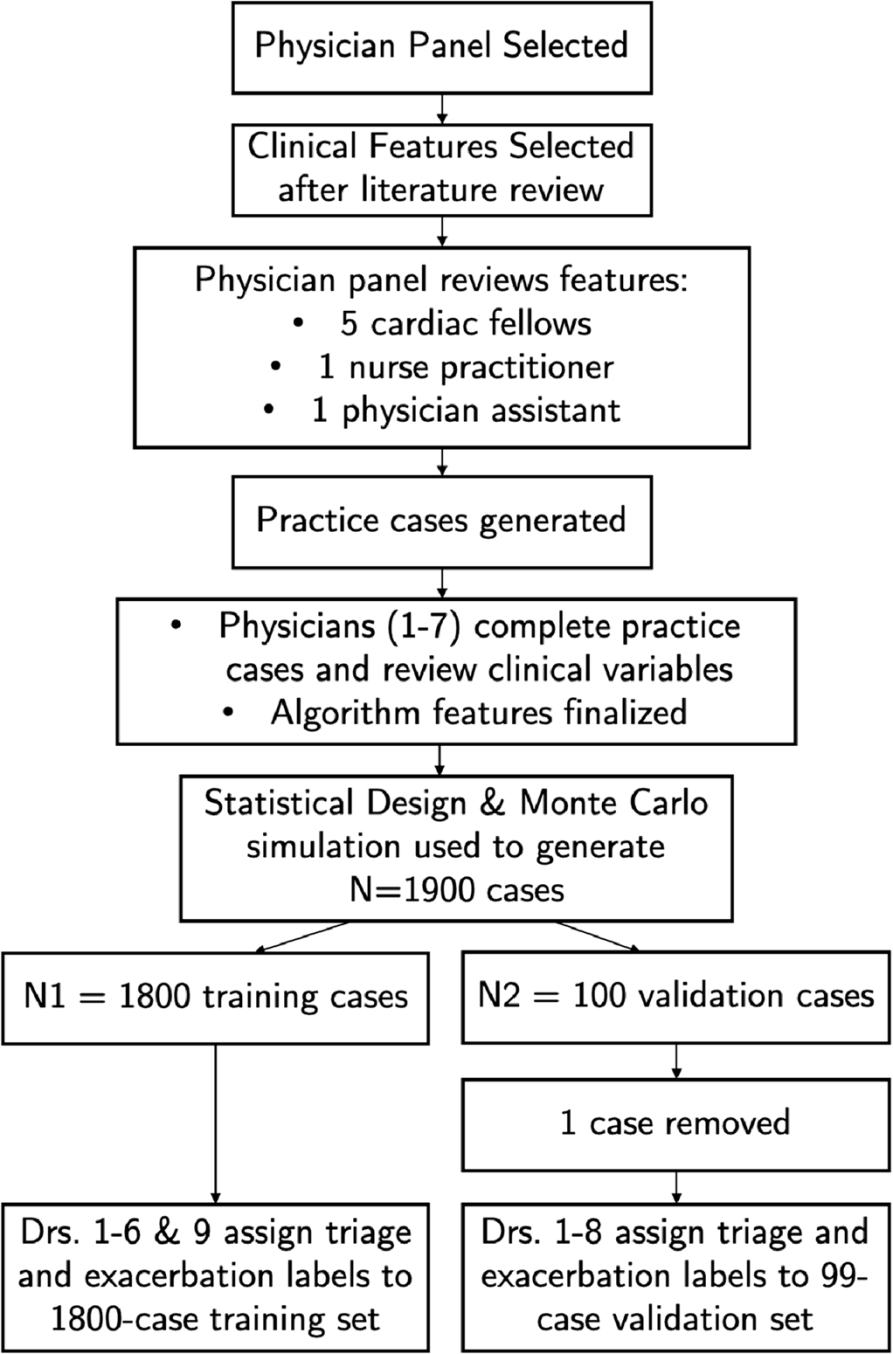
Description of the patient case generation process and splitting into training and validation data

Data was sent to scorers in 100-case batches. Cases that were used in the training were individually labeled by physicians, while cases used in the validation set included the opinion of all 8 previously mentioned specialists plus the algorithm. The process is depicted in Figure 1.

### Algorithm Training and Validation

**Figure 2:**
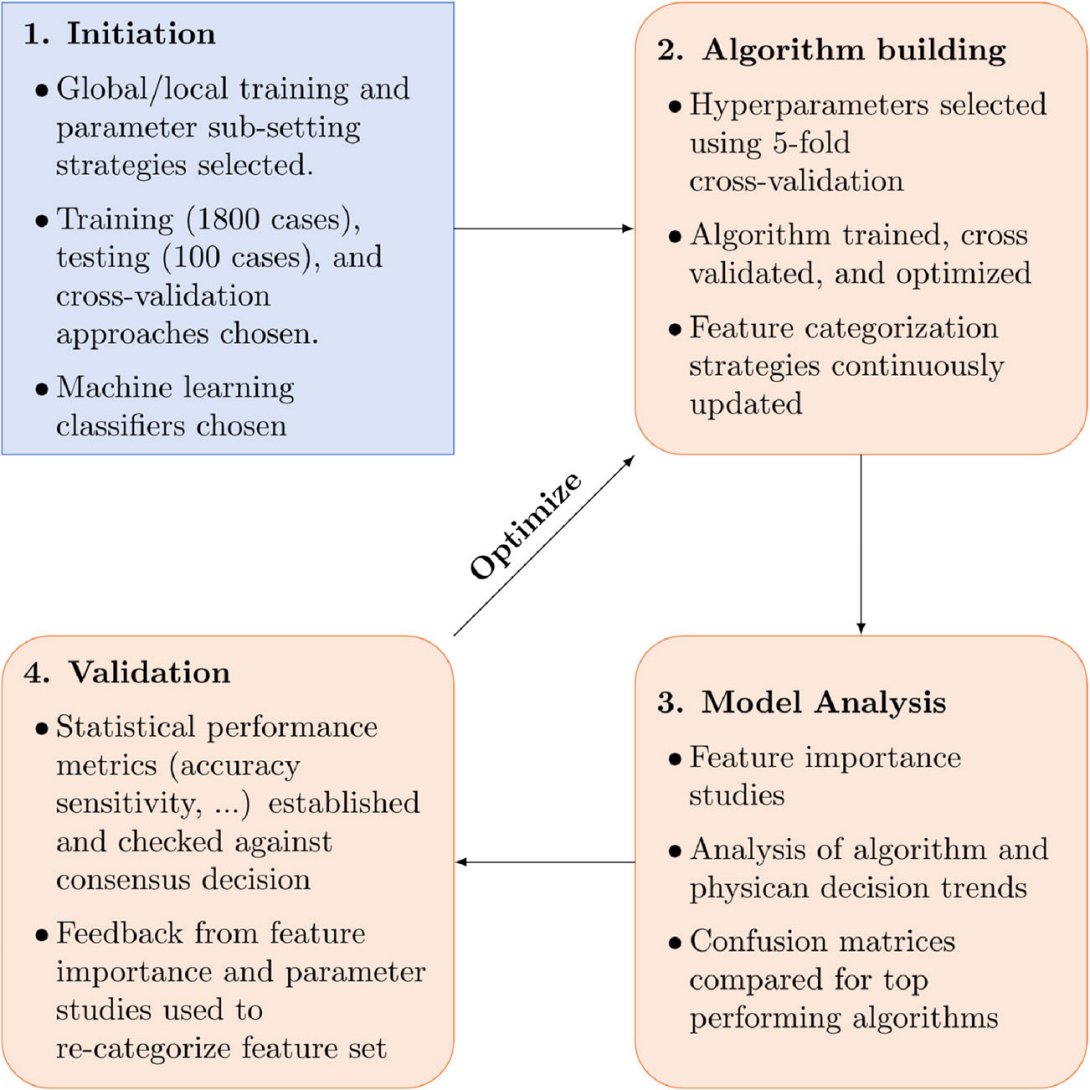
Algorithm training process

The strategy used to find the optimal prediction model is shown in Figure 2. This process was used in all triage, exacerbation, and recommended treatment decisions. Initially, several candidate supervised machine learning algorithms were selected including support vector machines, logistic regression, naive Bayes, linear discriminant analysis, K Nearest Neighbors, a variety of gradient boosted and ensemble decision tree methods, a multi-layer perceptron neural net, and several variants of such classifiers employing both soft and hard voting rules. Each classifier was run through an exhaustive grid search on the training set with 5-fold cross-validation. The top-performing algorithms of each class were selected based on how they performed when making predictions on the out-of-sample validation test.

The validation sets for triage, exacerbation, and treatment algorithms included consensus opinion on 100 clinically relevant patient cases scored by a panel of physicians and triage specialists. Each scorer and the algorithm were tested for how often their particular recommendation for a patient case matched the majority opinion. In cases of ties (rare events given the 9-member panel), the more conservative medical decision (higher/more serious category) was accepted as the correct one.

It is noteworthy that the 100 validation cases were removed from the case set before training which made them truly out-of-sample. Statistical measures of performance used in this study included:

$$ {\displaystyle \begin{array}{l}\mathbf{Classification}\ \mathbf{Accuracy}\ \left(\%\right)=\mathbf{ACC}=\frac{TC}{TC+ FC},\\ {}\begin{array}{l}\mathbf{ER}\ \mathbf{Sensitivity}={SENS}_{ER}=\frac{TP_{ER}}{TP_{ER}+{FN}_{ER}},\\ {}\begin{array}{l}\mathbf{ER}\ \mathbf{Specificity}={SPEC}_{ER}=\frac{TN_{ER}}{TN_{ER}+{FP}_{ER}},\\ {}\begin{array}{l}\mathbf{Positive}\ \mathbf{Predictive}\ \mathbf{Value}=\mathbf{PPV}=\frac{TP_{ER}}{TP_{ER}+{FP}_{ER}},\\ {}\begin{array}{l}\mathbf{Negative}\ \mathbf{Predictive}\ \mathbf{Value}=\mathbf{NPV}=\frac{TN_{ER}}{TN_{ER}+{FN}_{ER}},\\ {}\begin{array}{l}\mathbf{Confusion}\ \mathbf{Matrix}\ \mathbf{Proximity}\ \mathbf{to}\ \mathbf{Upper}\ \mathbf{Triangle}=\mathbf{UTP}=1-\frac{LT}{99},\\ {}\mathbf{Misclassifications}\ \mathbf{Greater}\ \mathbf{Than}\ \mathbf{One}\ \mathbf{Category}={E}_{G1}=\frac{C_{G1}}{99}\end{array}\end{array}\end{array}\end{array}\end{array}\end{array}} $$

where

*TC*: Total classifications matching the consensus
*FC*: Total classifications not matching the consensus
*TP*_*ER*_: Emergency classifications matching the consensus
*TN*_*ER*_: Non-emergency classifications matching the consensus
*FP*_*ER*_: Emergency classifications not matching the consensus
*FN*_*ER*_: Non-emergency classifications not matching the consensus
*LT*: Number of lower triangle entries in the confusion matrix
*C*_*G*1_: Number of triage misclassifications greater than 1 category

An additional set of analyses were done to subset circumstances in which patients did or did not require any type of medical attention. Here, the need for medical attention is defined as a situation in which the consensus specialist triage score is category 3 or 4. Categories 1 and 2 define the situations where no medical attention is required. The measures of performance used in this study are as follows:

$$ {\displaystyle \begin{array}{l}{ACC}_M=\frac{TC_M}{TC_M+{FC}_M},\\ {}\begin{array}{l}{SENS}_M=\frac{TP_M}{TP_M+{FN}_M},\\ {}\begin{array}{l}{SPEC}_M=\frac{TN_M}{TN_M+{FP}_M},\\ {}\begin{array}{l}{PPV}_M=\frac{TP_M}{TP_M+{FP}_M},\\ {}{NPV}_M=\frac{TN_M}{TN_M+{FN}_M},\end{array}\end{array}\end{array}\end{array}} $$

where

*TC*_*M*_: Total classifications matching the consensus view on the need or lack of need for medical attention
*FC*_*M*_: Total classifications not matching the consensus view on the need or lack of need for medical attention
*TP*_*M*_: Sum of category 3 and 4 classifications made on cases with a consensus triage of at least category 3
*TN*_*M*_: Sum of category 1 and 2 classifications made on cases with a consensus triage of at most category 2
*FP*_*M*_: Sum of category 3 and 4 classifications made on cases with a consensus triage of at most category 2
*FN*_*M*_: Sum of category 1 and 2 classifications made on cases with a consensus triage of at least category 3

## Results

### Algorithm Performance

#### Algorithm Feature Set

A large sample of potential features for exacerbation identification were used in this study. So as to prevent model overfit, the most relevant features were selected by applying a selection algorithm. The choice of algorithm was left as a hyperparameter to be optimized in model training. As an example, we present the reduced feature set chosen by the logistic feature selection method (implemented by [11]) for the triage prediction task in Table 1.

**Table 1:** All features used in the triage identification task

Category	Variable	Units
Patient profile	Gender	M/F-ON
Symptoms	Symptoms worse?	Y/N-ON
	Faint or dizziness	Y/N-ON
	Cough or wheezing	Y/N-ON
	Food intolerance	Y/N-ON
	Leg or stomach swelling	Y/N-ON
	Waking w/ dyspnea or chest pain	Y/N-ON
	Cold symptoms	Y/N-ON
	Diminished activity	Y/N-ON
	Chest pain	Y/N-ON
	Dyspnea	Y/N-ON
	Can’t get off bed or couch	Y/N-ON
	Can’t hold down food	Y/N-ON
Additional	Consumed high sodium food in past 24h	Y/N-ON
	Reduced appetite and can’t hold down food	Y/N-ON
	Taking additional diuretics for ≥ 7 days	Y/N-ON
	None	Y/N-ON
	O2 saturation	%-IR
	Weight	lb-IR
Vitals	Systolic blood pressure	mmHg-IR
	Heart rate	BPM-IR
	Resp rate	Breath/min-IR
	Temperature	°F-IR

*ON* categorical, *IR* continuous

### Algorithm Accuracy

**Figure 3:**
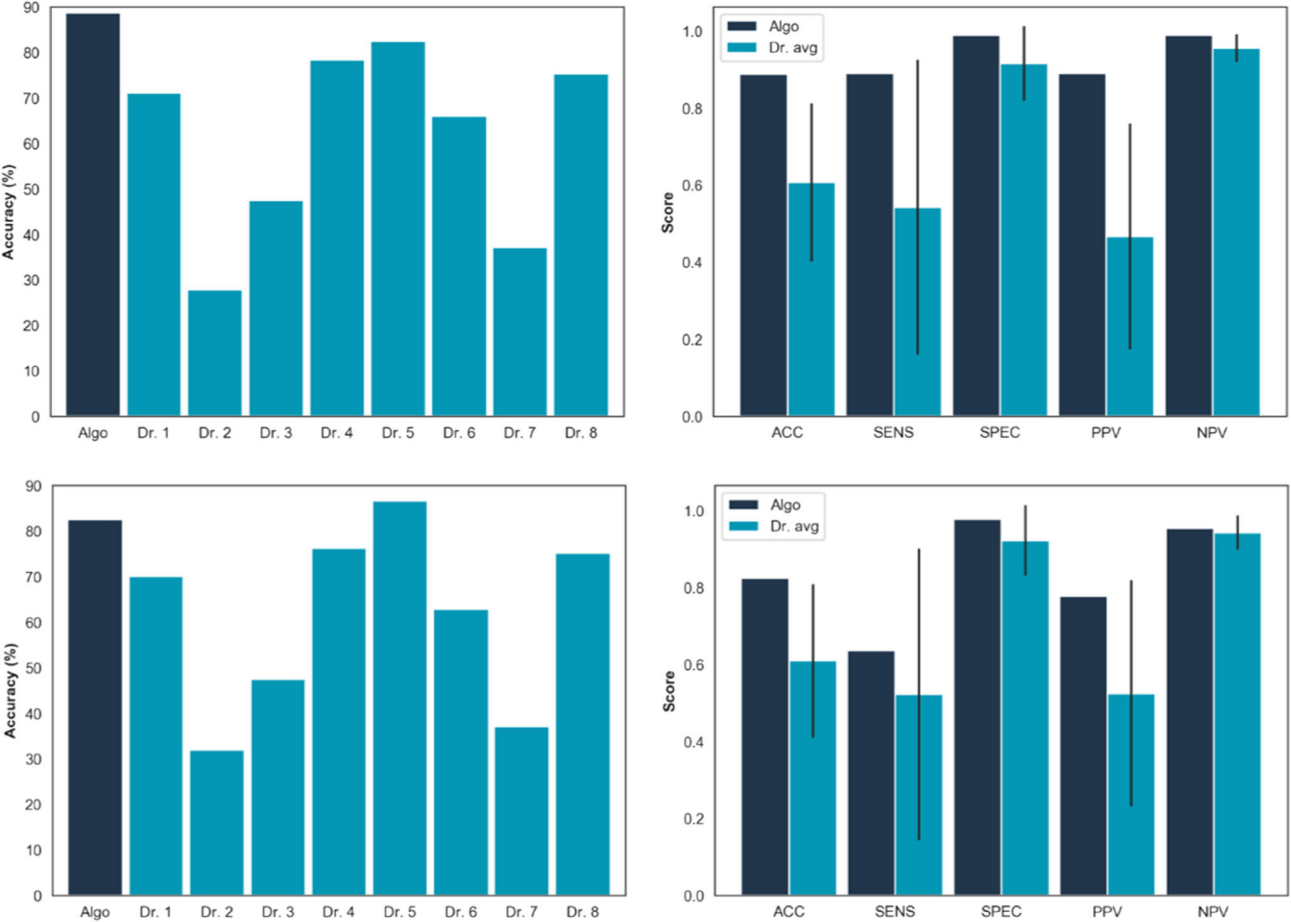
Top: Accuracy comparison of the algorithm and the individual physicians at predicting the validation set consensus for triage identification (left). Comparison of the major performance metrics between the algorithm and the average physician (right). The black line represents one standard deviation. Bottom: Comparison of the accuracy (left) and performance metrics (right) of the algorithm against the physicians when member votes are not included in assessing the accuracy. The black line represents one standard deviation

Algorithm performance was measured against a 100-case validation set, with ground truth being taken as the majority opinion of a panel of 9 specialists (experience and type of specialist are indicated in Table 4). We found that for each of triage, exacerbation, and treatment identification, the top-performing algorithm consisted of a combination of linear discriminant analysis and naive Bayes classifiers combined through a soft voting strategy. Major performance metrics are given in Eqs. (1)–(7). The top row in Figure 3 displays the accuracy of the algorithm against the accuracy of the individual doctors alongside the performance of the algorithm compared with the average doctor on each of the performance metrics.

For the triage class, the algorithm agreed with the consensus opinion in 89% of cases whereas the top doctor achieved an 83% accuracy. In exacerbation prediction, the algorithm again outperformed the leading physician with 94% accuracy compared with 91%. When recommending treatment, the algorithm reached a 68% agreement with the majority compared with 64% from the top physician.

The bottom row in Figure 3 shows an analogous plot to the row above but includes performance results in which the algorithm did not vote towards the consensus decision. The ground truth was thus taken to be the majority of just the physician decisions (a comparison that inherently offers a disadvantage to the algorithm). This test still found strong performance of the algorithm with it scoring second-placed in accuracy and retaining higher scores than the average physician in each of the performance metrics.

#### Confusion Matrix Analysis

**Figure 4:**
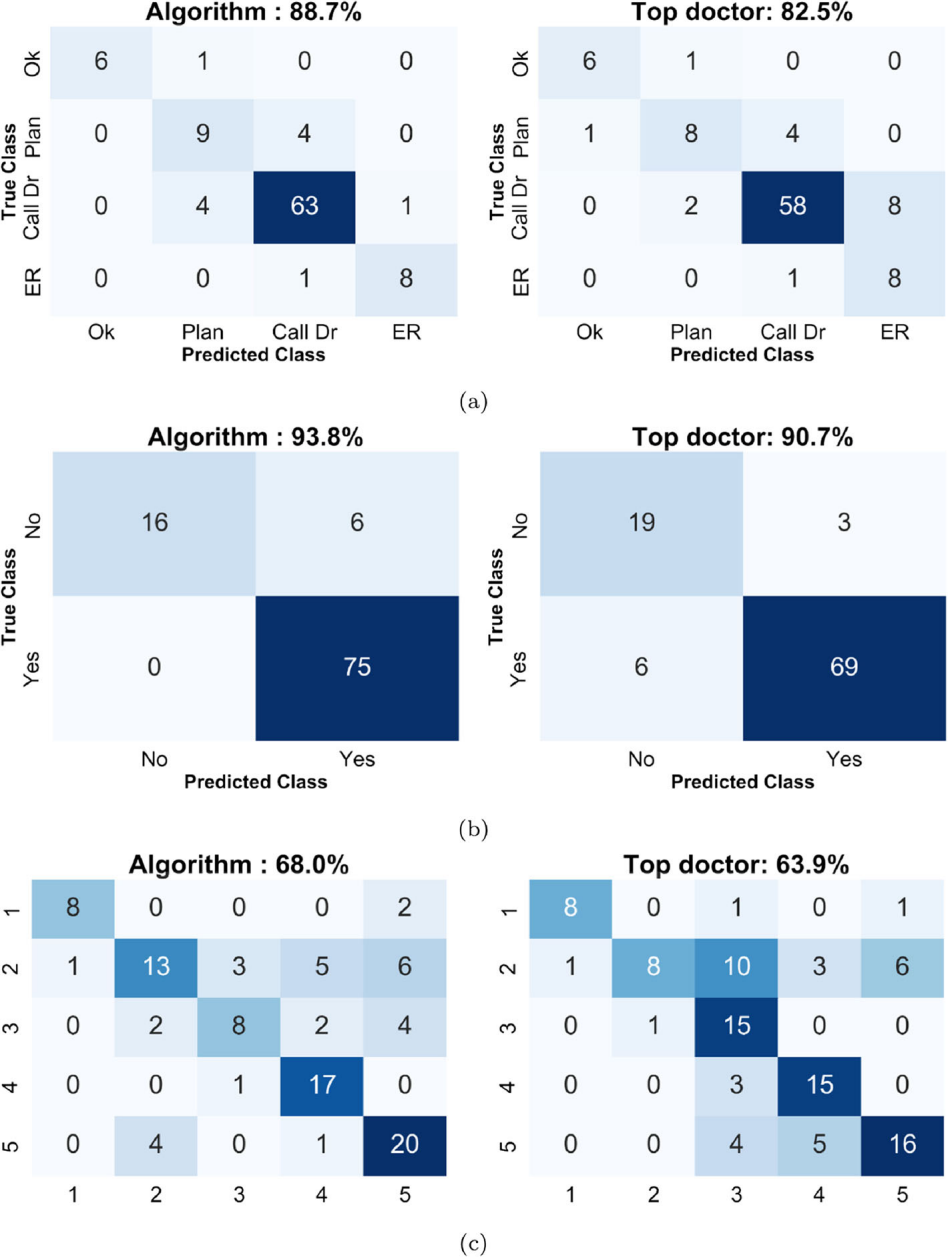
Confusion matrices of the algorithm and the top physician (in terms of accuracy) for **a** final triage, **b** exacerbation, and **c** recommended treatment detection

The confusion matrices for the algorithm against the top-performing doctor are displayed in Figure 4. These along with an evaluation of the metrics listed in Table 2 provide a comprehensive summary of algorithm performance compared with that of the physicians. When assigning triage categories, the algorithm achieved an 89% sensitivity in triaging to the ER and a 99% specificity in assigning a medical attention category. This is significantly better than the average doctor and better or equal to the top doctor in each category. The PPV achieved was 89% which is significantly better than the top doctor who had a 50% PPV due to a large over-prediction of ER cases.

**Table 2:** Statistical measures for all prediction tasks. Empty values exist in situations where the statistic is not applicable for that task

Metric	Triage		Exacerbation		Treatment	
	Algorithm	Top Dr	Algorithm	Top Dr	Algorithm	Top Dr
**ACC**	88.7	82.5	93.8	90.7	68.0	63.9
**SENS**	88.9	88.9	100.0	92.0	–	–
**SPEC**	98.9	90.9	72.7	86.4	–	–
**PPV**	88.9	50.0	92.6	95.8	–	–
**NPV**	98.9	98.9	100.0	76.0	–	–
*ACC*_*M*_	91.8	93.8	–	–	–	–
*SENS*_*M*_	94.8	97.4	–	–	–	–
*SPEC*_*M*_	80.0	80.8	–	–	–	–
*PPV*_*M*_	94.8	94.8	–	–	–	–
*NPV*_*M*_	80.0	88.9	–	–	–	–
**UTP**	94.8	95.9	–	–	–	–

It is worth noting that if we remove the algorithm’s “opinion” from the validation set consensus, the algorithm still achieves the top performance in triage identification with 83% prediction accuracy compared with 77% from the top-performing physician. This finding indicates that the algorithm maintains top performance even when subjected to a test that unfairly advantages the medical specialists.

From the confusion matrix of treatment prediction, it is important to remember that class ‘5’ corresponds to a finding that the specialist was not comfortable making a treatment recommendation”.

#### Feature Importance

In Table 3, we indicate the 10 most relevant features when predicting the triage and exacerbation categories. In both cases, the features selected by the algorithms are assumed to be the most discriminating when diagnosing HF exacerbation events.

**Table 3:** Top 10 most important features, as ranked by the top performing algorithm, for the triage and exacerbation identification tasks

Triage	Exacerbation
No current symptoms	Weight gain (7, inf]
Current O2Sat (87, 89]	Waking at night with dyspnea or chest pain
No worsening of symptoms	Leg or stomach swelling
Can't get out of bed or off couch	Weight gain\_(-inf, 1]
O2Sat gain (-inf, 1]	No worsening of symptoms
Weight gain (6, inf]	Cold symptoms
Cold symptoms	Dyspnea
Current O2Sat (-inf, 87]	Weight gain (2, 7]
Chest pain	Current HR (95.0, 102.0]
Dyspnea	No current symptoms

**Table 4:** List of medical specialists, their affiliations, and their contribution to this study process

Institution	Title	Certification BC = board certified BE = board eligible	Algorithm feature feedback	Training data case scoring	Validation data case scoring
Kaiser Permanente, Oakland, Ca	Practicing Cardiologist	BC: Internal Medicine BC: Cardiology	X	X	X
Duke University Durham, NC	Cardiology Fellow (year 3)	BC: Internal Medicine BE: Cardiology	X	X	X
Alaska Heart Anchorage, AK	Practicing Cardiologist	BC: Internal Medicine BC: Cardiology	X		X
Duke University Durham, NC	Cardiology Fellow (year 3)	BC: Internal Medicine BE: Cardiology	X		X
Alaska Heart Anchorage, AK	Physician Assistant	PA-C		X	X
Duke University Durham, NC	Interventional and Structural Cardiology Fellow (year 3)	BC: Internal Medicine BE: Cardiology	X	X	X
University of Kansas Kansas City, KA	Nurse Practitioner	DNP		X	X
Duke University Durham, NC	Cardiovascular Disease Fellow (year 5)	BC: Internal Medicine BC: Pulmonary Disease	X	X	X
Duke University Durham, NC	Cardiology Fellow (year 3)	BC: Internal Medicine BE: Cardiology PhD: Physiology and Biophysics		X	

### Validation Set

#### Physician Decision-Making Trends

**Figure 5:**
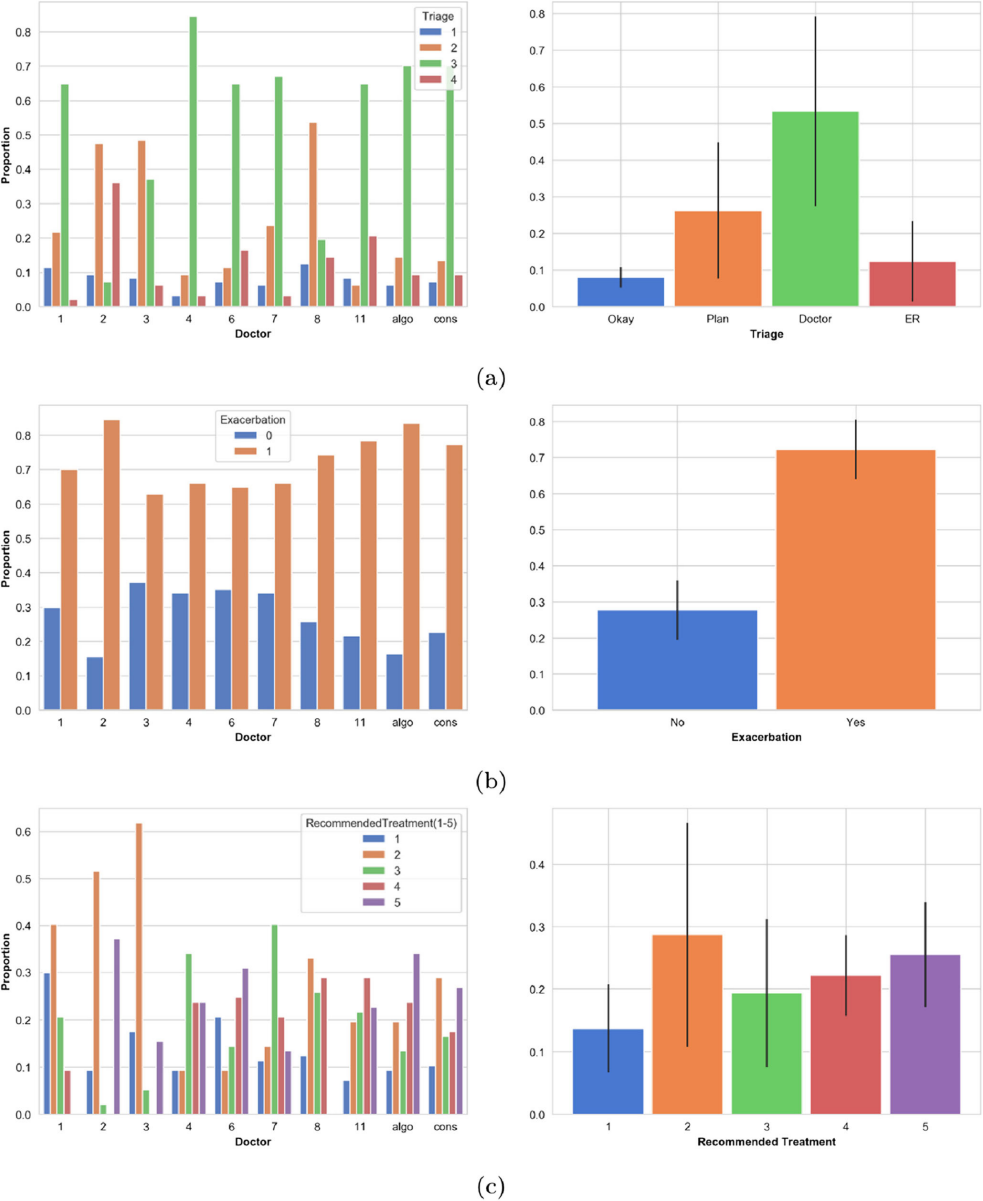
Distribution of decisions for each physician (left) and averaged physician decision distribution (right), the black line represents 1 standard deviation about the mean. **a** Triage, **b** exacerbation, **c** recommended treatment

In Figure 5, we plot the distribution of decisions made by each physician (left charts) alongside the averaged physician distributions with error bars that denote 1 standard deviation from the mean, for each of the target variables. We see significant variation in opinion between physicians; for example, doctor 4 believes 87.5% of cases warrant medical attention but doctor 8 only 34.0%. We note that only 4.0% of total triage assignments were ever more than one triage category away from the consensus decision, so decisions are very rarely made far from the average. We also observed wide variation in apparent definitions of exacerbation; for instance, in only 45% of the cases that doctor 2 determines as experiencing an exacerbation do they also triage a medical attention category. Conversely, 100% of the cases doctor 4 determines as exacerbating are also given a medical attention triage label. We finally note that 8.5% of cases triaged to a medical attention category were not predicted to be experiencing an exacerbation, suggesting that physicians may have thought an alternate diagnosis was driving symptoms.

### Robustness of Validation Set Consensus

**Figure 6:**
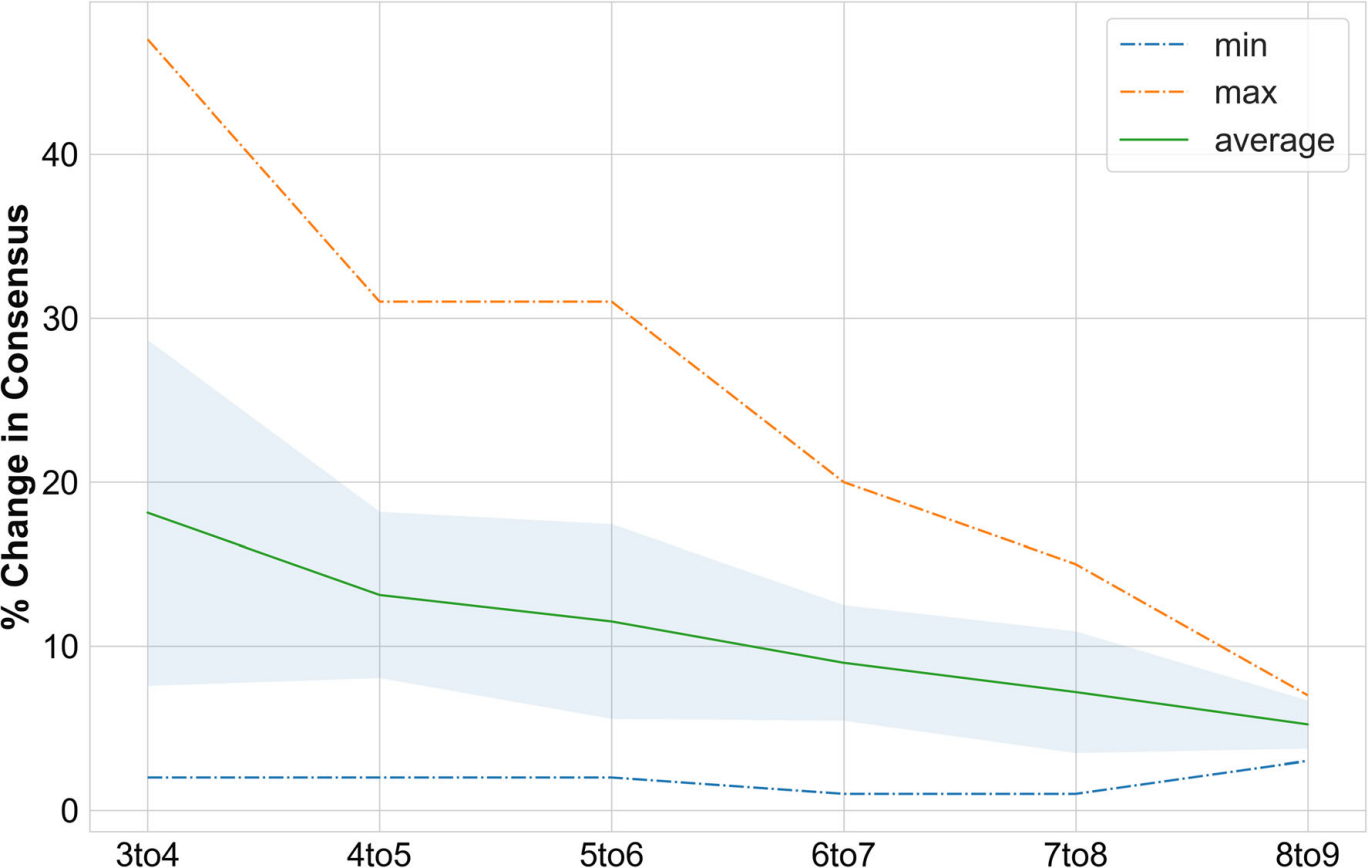
Plot of % of triage cases in the validation set that change consensus decision as additional doctors (plus the algorithm) are added to the validation panel. The shaded region around the mean represents one standard deviation. The average change when the panel increases from 8 to 9 members is 5.2%

In Figure 6, we plot information regarding the number of cases that change when an additional doctor is added to physician panels of differing sizes. For example, the data at the point “4 to 5” refers to the number of validation case consensus opinions that change when the panel size grows from 4 to 5 physicians. We compute the % change for all possible combinations in the data set and plot the max change, min change, and mean change with associated standard deviation. A convergence of consensus opinion is observed as opinion as more doctors are added with the change being just 5.2% when transitioning from 8 to 9 doctors, 9 being the total number of opinions in the final validation set.

## Discussion

The clinical need for novel approaches to at-home HF care is eminently apparent. At present, technological solutions have generated a great deal of interest in the management of HF and HF-related conditions. Examples include the early detection of atrial fibrillation from smartphone sensors [12], ambulatory pulmonary artery pressure monitoring that can detect worsening HF signs [13], and ECG monitoring from iPads to detect atrial fibrillation [14, 15]. Telehealth applications also exist and have grown in use in the wake of the 2020 Covid-19 pandemic [16–18], but they require patient self-identification of health deterioration before initiation, and long-term adoption is still uncertain in the current healthcare reimbursement and regulatory landscapes [4, 19–21].

Sensors that record physiological inputs (like vital signs or activity) are being increasingly embedded in applications for the purpose of remote patient monitoring, telehealth, and chronic care management [4]. A known drawback of these approaches, however, is that pure vital sign monitoring is known to yield false positives and negatives in the absence of other contextual information like patient profile, symptom, and circumstance data [22–24]. Machine learning models like those offered in this study and other interpretation algorithms have the power to make remote monitoring devices actionable and accurate while obviating the need for expensive continuous at-home nurse care. For example, prediction algorithms could be accessed via hardware and software agnostic APIs (application programming interfaces) or SDKs (software developer kits), and deliver interpretation of a patients’ signs and symptoms through any number of existing hardware devices (mobile phones, tablets, computers, monitoring devices, etc.) or software (Payroll, HR, Identity Management, etc.). Algorithms could receive health data from a caregiver, patient, and/or measurement device and return assessments of potential exacerbations in real time.

The algorithms produced in this study were validated by comparing performance predictions on a representative set of patient scenarios to the opinions of 8 board-certified medical professionals. Given the current standard of care emphasis on internists, triage nurses, and other medical professionals providing remote triage of health escalations, the aforementioned validation procedure constitutes a meaningful performance standard. Analysis of the algorithm performance and the physician provided data showed (1) the algorithm exhibited exceptional performance when compared to individual physicians at assessing the likelihood that a patient is experiencing an exacerbation and identifying the appropriate consensus triage category and (2) the algorithm triaged in favor of the safety of the patient, when disagreeing with consensus, more often than individual specialists.

The results in this study show that algorithmic support can be provided to patients—in line with that of a consensus panel of physicians—from health characteristics that are readily available at home.

While the prediction models presented in this study are not meant to be a substitute for physician examinations, they do provide a methodology for deploying at-home decision support in a variety of consumer-facing devices which can provide patients with important insights and direction including mobile/web apps, continuous monitoring devices, and smart medication deployment tools. The patient would receive timely interventions and decision support in instances of clinically significant health deterioration. It should also be emphasized that the methods employed in this study are generalizable to other chronic illnesses in which patient profile, symptom, and vital sign data can be plausibly captured and relevantly applied to exacerbation detection.

## Limitations

While the algorithms presented in this study exhibited very strong performance when predicting on the out-of-sample validation set, ultimately the methodology of its training relied on expert opinion for assignment of case labels. This approach is limited by the inherent variation and accuracy of the experts who labeled the cases along with the relatively small number of individual experts. On the other hand, this observed variability in practice points to the necessity of improved triage tools. Also, thus far, the HF triage algorithms have been both developed and tested on hypothetical patient cases. While the use of simulated data is a primary strength of this study’s modeling approach given its importance in developing truly representative prediction algorithms, an additional level of validation could be conducted to compare the prediction of the algorithms in real-world clinical setting with a set of physicians actively triaging the same set of patients. This exercise, in addition to efficacy trials to show the therapeutic benefit of these algorithms when deployed in a consumer facing applications, has been conducted for chronic lung disease patients in [25] and is slated for future studies.

Finally, the black-box nature of nested machine-learning classifiers makes the decision-making logic in triage recommendations difficult to interpret. The feature importance studies previously discussed shed light on which patient variables most influence the final outcome, but ultimately, the inherent complexity and interactions of the feature set make it difficult to give a simple, linear causal explanation of the algorithm output based on the inputted features.

## Conclusion

This study has shown that a machine-learning approach to triaging patients with HF is a viable and accurate method of facilitating at-home triage and exacerbation self-identification when compared to individual heart specialists. The machine-learned triage approach in this study performed favorably when compared to individual heart specialists in a broad range of statistical performance measures in both exacerbation identification and severity assessment. Unlike existing paper checklist type tools, the models incorporated the baseline medical health of the patient in a way that robustly accounted for the complex interactions of patient health variables. This serves in contrast to existing deterministic guidelines and mobile applications. Moreover, the presented classification analysis showed that the algorithms generally erred in favor of the patient’s safety and showed strong performance in triaging emergency scenarios when compared to heart specialists. Finally, the algorithm prediction accuracy exceeded all individual specialists in predicting the consensus opinion on the presence of an exacerbation, the appropriate triage category, and the appropriate responsive medication category in a representative set of patient cases. The presented methodologies can also be generalized for remote assessment of infection risk and health deterioration associated with numerous other viral and chronic illnesses; the specific clinical features of prediction models would certainly vary across disease spaces, but the core data generation, machine learning, and validation strategies employed in this study could be adapted to other applications.

## Supplementary Information

ESM 1 (DOCX 14 kb)
