# Supplementary material for: A Machine Learning Methodology for Identification and Triage of Heart Failure Exacerbations
Source: J Cardiovasc Transl Res. 2021 Aug 28;15(1):103–15. doi: 10.1007/s12265-021-10151-7 (PMC8397870; doi:10.1007/s12265-021-10151-7)
Supplement: Supplementary file 1 — (DOCX 14 kb) [file 12265_2021_10151_MOESM1_ESM.docx]

**Supplementary Information**

Here we present further details regarding the machine learning training process including the models considered, hyperparameters optimized, and final algorithm pipelines for each task. Full code to reproduce the models seen in this paper is available at [***https://github.com/jambo6/jctr_chf_code***](https://github.com/jambo6/jctr_chf_code)***.***

Hyperparameters were optimized using 5-fold cross-validation with the top performing hyperparameter set on the average out-of-sample score being used to train the model on the full training data and evaluated on the validation set. The complete set of tunable parameters is:

- Choice of classifier. Considered classifiers were: Logistic Regression, Linear Discriminant Analysis, Naive Bayes, Support Vector Machine, Random Forest Classifier, and XGBoost.
- Hyperparameters of the chosen classifier.
- Cases marked by doctors as ‘uncertain’.
- Cases marked by doctors as ‘unrealistic’.
- Feature selection method (one of ‘Logistic’, ‘Lasso’, or ‘Boruta’)

Final choices of hyperparameters and trained models can be found in the github repository.

The top performing model choices for each of the prediction were:

- **Triage:** Naive Bayes
- **Exacerbation:** Logistic regression
- **Recommended treatment:** A combined Naive Bayes and Logistic Regression classifier.
